# Optimizing Ovarian Stimulation for IVF in PCOS Patients: A Novel Day 1 GnRH Antagonist Protocol

**DOI:** 10.3390/jcm14165901

**Published:** 2025-08-21

**Authors:** Sudarsan Ghosh Dastidar, Biswanath Ghosh Dastidar, Ratna Chattopadhyay, Chandan Chakraborty

**Affiliations:** 1 GD Institute for Fertility Research, 36 A/1 S.P. Mukherjee Road, Kolkata 700025, West Bengal, India; 2 Department of Obstetrics and Gynecology & GDIFR-IPGMER Center of Excellence (COE) in Assisted Reproductive Technology (ART), Institute of Post-Graduate Medical Education and Research (IPGMER) & SSKM Hospital, 244 AJC Bose Road, Kolkata 700020, West Bengal, India; 3 Institute of Reproductive Medicine, DD-18/5/1, Sector-1, Salt Lake, Kolkata 700064, West Bengal, India; 4 National Institute of Technical Teachers’ Training and Research, Falguni Road, FC Block, Sector 3, Salt Lake, Kolkata 700106, West Bengal, India

**Keywords:** ovarian stimulation (OS), GnRH antagonist, luteinizing hormone (LH), pituitary suppression, polycystic ovarian syndrome (PCOS), in vitro fertilization (IVF) outcome

## Abstract

**Objectives**: Gonadotropin-releasing hormone (GnRH) antagonist protocols are preferred in polycystic ovary syndrome (PCOS) patients undergoing in vitro fertilization (IVF) as they provide the best combination of flexibility, acceptable outcomes, and safety. Numerous studies have compared outcomes between GnRH agonist long protocol and standard flexible antagonist protocol. However, there are scant studies investigating the effectiveness of antagonist administration from day 1 of ovarian stimulation in PCOS patients. **Methods**: We performed a retrospective cohort study to compare laboratory and clinical outcomes in IVF between standard flexible day 5/day 6 versus day 1 GnRH antagonist protocol in PCOS patients. **Results**: Our data indicates significantly superior oocyte yield and top-quality embryo proportion in patients with antagonists from day 1. Cumulative clinical pregnancy rates also tended to be superior in this group. **Conclusions**: Our findings indicate that administration of GnRH antagonists from day 1 of stimulation in PCOS patients undergoing IVF may lead to superior results.

## 1. Introduction

Polycystic ovarian syndrome (PCOS) is the leading cause of anovulation globally, impacting up to 8 out of 10 women with this condition [1]. It has been estimated that up to 9–18% women of reproductive age suffer from PCOS [2]. PCOS is widely diagnosed in clinical practice based on the 2003 Rotterdam Consensus Criteria, with a diagnosis made if any two features out of oligo-anovulation (irregular menstrual cycles), clinical or subclinical/biochemical hyperandrogenism, and polycystic ovarian morphology (PCOM) on ultrasound are present [3]. The Androgen Excess and PCOS Society (AEPCOS) criteria for diagnosing PCOS focuses on hyperandrogenism [4]. A woman is diagnosed with PCOS as per AEPCOS guidelines if she exhibits clinical or biochemical hyperandrogenism, plus signs of oligo-anovulation or PCOM on ultrasound. PCOS is associated with comorbidities such as obesity, dyslipidemia, infertility, and metabolic syndrome, and long-term sequelae such as diabetes mellitus, cardiovascular disease, and endometrial cancer [5,6,7].

At the systemic level, the pathophysiology of PCOS is orchestrated by an interplay of endocrinologic and metabolic abnormalities. These include aberrant gonadotropin secretion patterns with a shift of balance toward elevated luteinizing hormone (LH) levels and insulin resistance leading to compensatory hyperinsulinemia, both of which directly and indirectly lead to clinical or subclinical hyperandrogenemia. At the tissue level, PCOS is also characterized by local hyperandrogenism in the ovaries. A disturbed intra-ovarian microenvironment together with systemic impairments leads to the arrest of the development of preantral follicles resulting in the characteristic PCO morphology. At the ovarian level, follicles in PCOS patients also exhibit relative resistance to the stimulatory effect of follicle-stimulating hormone (FSH). Follicular resistance to FSH signaling in PCOS may be intrinsic to the disease, secondary to intra-ovarian hyperandrogenism, and consequent to elevated levels of anti-Mullerian hormone (AMH) secreted by the growing cohort of preantral follicles [8]. Together, these mechanisms result in impaired cyclic development and arrest of follicles in PCOS [9].

Elevated serum LH appears to be a key factor leading to increased AMH levels in PCOS patients. Acting synergistically with elevated serum insulin, LH promotes AMH synthesis both directly and indirectly by increasing androgen production from ovarian theca cells [10,11,12]. At the level of the hypothalamic–pituitary–ovarian (HPO) axis, PCOS patients have been shown to exhibit increased LH pulse amplitude as well as pulse frequency, which traces back to impairments in the hypothalamic GnRH pulse generator.

GnRH agonists have been used over the past few decades in ovarian stimulation (OS) regimens to optimize in vitro fertilization (IVF) outcomes by controlling premature LH surges, preventing premature ovulation, and achieving predictable ovarian response. GnRH antagonists are currently preferred, especially in PCOS patients. Advantages associated with the use of GnRH antagonists include quick and reversible pituitary suppression, correction of the deranged internal hormonal milieu, and reduction in the incidence of ovarian hyperstimulation syndrome (OHSS). Use of an antagonist protocol also facilitates maturation triggering with GnRH agonists. Employing this strategy, preferably followed by elective embryo cryopreservation and frozen embryo transfer (FET), further reduces the risk of OHSS, avoids luteal phase defect (LPD), and improves pregnancy rates [13].

It has been conclusively demonstrated that IVF outcomes with GnRH antagonists are comparable to those with GnRH agonists, with the added advantage of a significantly reduced risk of OHSS [14,15,16,17,18,19,20,21]. In addition, it has been shown that use of antagonists in OS regimens is associated with a reduction in the duration of stimulation and total dosage of gonadotropins used in IVF cycles compared to GnRH agonists [22,23,24,25].

Numerous studies have compared IVF outcomes between patients undergoing OS using a GnRH agonist versus flexible day 5/day 6 antagonist protocols. However, there is scarce data comparing the use of GnRH antagonists from day 1 of IVF stimulation versus standard flexible antagonist administration from day 5/6 in PCOS patients undergoing IVF cycles, in terms of possible impact on quantitative and qualitative laboratory and clinical outcome parameters such as number of oocytes retrieved, fertilization rate resulting in optimal 2PN embryos, number of embryos formed, proportion of top-quality embryos achieved, and pregnancy rates. Theoretically speaking, if it is assumed that GnRH antagonist-mediated suppression of elevated serum LH levels in PCOS patients during OS is likely to improve the impaired internal hormonal milieu and thereby optimize IVF outcomes, it stands to reason that it may be of benefit to administer antagonists from day 1 of stimulation itself.

Indeed, differences in serum LH levels have been reported in the follicular phase of IVF stimulation between patients undergoing OS by agonist protocol and GnRH antagonists from day 1 of stimulation [26]. Unfortunately, a three-arm study comparing early antagonist use from day 1 of stimulation versus flexible antagonist administration from day 5 or day 6 versus agonist protocols was not designed to evaluate the qualitative aspects of laboratory parameters but reported that the number of oocytes obtained was similar between the three protocols and that clinical pregnancy rates showed a higher trend in the early antagonist group, although without statistical significance [27].

Thus, there is a strong scientific rationale and need to investigate whether the administration of GnRH antagonists from day 1 of IVF stimulation may result in better laboratory and clinical outcomes compared to the standard flexible protocol of administering GnRH antagonists from day 5 or day 6 of OS. 

We presented our experience of achieving favorable IVF outcomes using GnRH antagonists from the first day of ovarian stimulation in PCOS patients in a case series at the Annual Congress of the American Society for Reproductive Medicine (ASRM) [28]. Since then, we have been employing this strategy in our routine clinical practice for OS in selected PCOS patients undergoing IVF cycles. The present retrospective cohort study was designed to compare the laboratory and clinical outcomes of IVF in PCOS patients using GnRH antagonists from day 1 of stimulation versus the standard protocol of flexible antagonist administration from day 5 or day 6 of OS.

## 2. Materials and Methods

### 2.1. Study Design, Setting, Ethics, Sample Size Determination, and Informed Consent

This retrospective cohort study was carried out at a leading fertility research institute associated with a university medical college and tertiary-care multi-specialty hospital, in collaboration with a leading private fertility institute. Following institutional ethical clearance, PCOS patients (as defined by the Rotterdam Consensus Criteria, 2003) who had undergone IVF using antagonist cycles from January 2025 to June 2025 were included in the study. A sample size calculation revealed that to be powered at 80% to test for differences between the two groups at a significance level of 0.05, with an effect size d = 0.8 (Cohen’s d), each group required at least 24.5 (rounded off to 25) participants. Informed, written consent for participation was obtained from all patients who agreed to have their IVF cycle data analyzed as part of this study. Moreover, all patients provided unlimited permission for the publication of the study results in all formats (print, electronic, online) as long as their identity was masked, anonymity strictly safeguarded, and personally identifiable data kept confidential.

### 2.2. Exclusion Criteria and Outcome Measures

Patients with a history of Grade III or Grade IV endometriosis, as well as patients with concurrent male factor infertility, were excluded from the study. To ensure interpretability of results, only conventional IVF cycles were included, and all cycles with intra-cytoplasmic sperm injection (ICSI) were excluded from the analysis. The clinical outcome measure was cumulative clinical pregnancy rate (CPR) over a maximum of 3 embryo transfer cycles (fresh and/or frozen). The laboratory outcome measures were oocyte yield, proportion of oocytes fertilized by conventional IVF (fertilization rate), and proportion of top-quality embryos obtained.

### 2.3. Clinical Protocol

**Figure 1:**
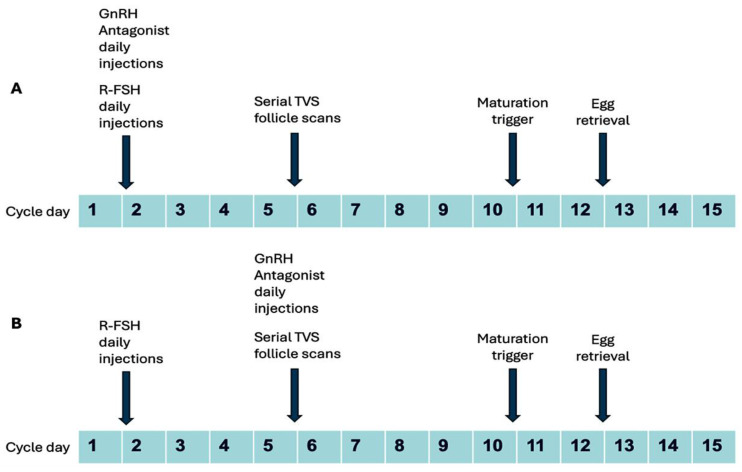
**Schematic depiction of typical ovarian Stimulation protocols.** In Group 1, GnRH Antagonist (Cetrorelix acetate 0.25 mg daily injections) was started from day 1 of stimulation concurrently with r-FSH (**A**); In Group 2, r-FSH was started on cycle day 1or 2 and flexible GnRH antagonist administration initiated from day 5 or 6 of stimulation (**B**).

Most patients had undergone ovarian stimulation with a starting dose of 100–200 IU of recombinant follicle-stimulating hormone (r-FSH/Follitropin alpha, Folisurge, Intas Pharmaceuticals Ltd., Ahmedabad, India) from day 1 or day 2 of the menstrual cycle (going up to 225 IU in a few patients), with dose adjustment conducted following serial serum estradiol (E2) estimation and follicle tracking by transvaginal sonography (TVS) from day 5 or day 6 of stimulation. Patients who had been administered a GnRH antagonist in the form of 0.25 mg Cetrorelix acetate daily injections (Cetrolix, Intas Pharmaceuticals Ltd.) early from day 1 of stimulation were considered as Group 1 (Test cohort), and patients who had received 0.25 mg Cetrorelix acetate daily injections from day 5 or day 6 as per the standard flexible protocol were considered as Group 2 (Control cohort). The trigger for final oocyte maturation was given with 250 mcg recombinant human chorionic gonadotropin (r-hCG, Ovitrelle, Merck Specialities Pvt. Ltd., Mumbai, India) or bolus GnRH agonist administration using a total of 0.1 × 2 mg triptorelin (Decapeptyl, Ferring Pharmaceuticals, Saint-Prex, Switzerland) when at least 2–3 follicles measured 17–18 mm or more on TVS. Egg retrievals were performed 34–36 h after the maturation trigger by the standard TVS-guided protocol. Typical stimulation protocols are shown schematically in Figure 1.

### 2.4. Laboratory Protocol

COCs were inseminated by conventional IVF in all patients using 15,000–20,000 sperm per COC. Fertilization was confirmed by documenting the 2PN stage 17–18 h post-insemination (hpi). Embryos were cultured under standard IVF embryo culture conditions in conventional benchtop incubators (Origio, CooperSurgical, Trumbull, CT, USA). On day 3 following fertilization, all embryos were graded by morphological analysis of still images under an inverted optical microscope (Olympus Corporation, Tokyo, Japan), as per ESHRE-ALPHA Scientists Istanbul Consensus Criteria [29]. Following embryo grading on day 3, the majority of embryos were cryopreserved across both groups for subsequent frozen–thawed transfers. However, fresh embryo transfers were performed in certain patients based on various factors (clinical, number of oocytes/embryos, patient preference, etc.). The majority of transfers across both groups were blastocyst transfers, although a few patients were transferred cleavage-stage embryos as well. Cryopreservation of embryos was performed following standard vitrification protocol using Cryotop vitrification kits (Kitazato Corporation, Shizuoka, Japan).

### 2.5. Embryo Transfer and Cryopreservation

All embryos were transferred under abdominal ultrasound guidance using Wallace embryo transfer catheters (CooperSurgical, USA). All ETs and FETs were performed by the same clinician. For FETs, embryo warming was performed by standard protocols using Kitazato kits, and transfers were conducted in artificially prepared cycles using hormone replacement therapy (HRT) with estradiol valerate (Progynova 4–8 mg per day, Bayer-Zydus Pharma Ltd., Mumbai, India), followed by intramuscular progesterone injections (Gestone, 50 mg per day, Ferring Pharmaceuticals), once endometrial thickness reached 7 mm or more.

Patients with both cleavage-stage embryo transfers and blastocyst transfers were included when analyzing pregnancy outcomes. A maximum of 2 embryos were transferred at any single embryo transfer. Fresh transfers were only performed in cycles triggered with r-hCG.

### 2.6. Luteal Support and Documentation of Pregnancy

Luteal support was given as per standard protocol with 50 mg intramuscular progesterone injections for 2 weeks followed by the application of 8% micronized progesterone (Crinone 8%, Merck Ltd.) vaginally till 10 weeks of gestation. Biochemical pregnancy was documented by serial serum beta-hCG estimations 48 h apart around 2 weeks following embryo transfer, followed by confirmation of a viable clinical pregnancy by transvaginal sonographic demonstration of fetal heartbeat at 5–6 weeks of gestation.

### 2.7. Retrospective Cohort Analysis: Embryo Grading

Oocyte yield, fertilization rate, top-quality embryo rate, and pregnancy rates were analyzed for the two cohorts retrospectively. For retrospective cohort analysis of top-quality embryo rate, day 3 cleavage-stage embryo images were used as embryos had been vitrified at that stage in many cases and were still in storage. Each embryo was graded independently by 3 different embryologists in a blinded fashion. Embryologists who graded the embryos were not aware of the group allocation of the patient or of the scores given by the others. Embryo grading was performed as per the updated ESHRE-ALPHA Scientists Istanbul Consensus Criteria. Any differences in grade were resolved by consensus.

### 2.8. Statistical Analysis

Numerical data was tabulated in Microsoft Excel sheets and descriptive summary statistics were attained. Statistical analysis of data was performed using Python version 3.11.13 for analytical statistics. An independent samples Student’s *t* test was used to estimate differences between means, and a Z test was used to estimate differences between proportions/rates, with any difference considered to be statistically significant when *p* < 0.05.

### 2.9. Final Data Presentation

A total of 100 patients’ data was analyzed. Baseline parameters such as age, body mass index (BMI), AMH, thyroid-stimulating hormone (TSH) levels, and average duration of stimulation were found to be comparable between the two groups, as shown in Table 1.

Cumulative pregnancy data was analyzed over a maximum of 3 transfer cycles—1 fresh cycle (when performed) and up to a maximum of 2 frozen transfers. Outcomes were compared between Group 1 (day 1 antagonist, n = 45) and Group 2 (day 5/day 6 antagonist, n = 55). Pregnancy data is presented only for 97 patients, as 3 patients in Group 1 did not have embryo transfers. Miscarriage rates (MRs) and ongoing pregnancy rates (OPRs) could not be calculated because some patients are currently pregnant below 10 weeks of gestation.

## 3. Results

### 3.1. Laboratory Outcome Measures

**Figure 2:**
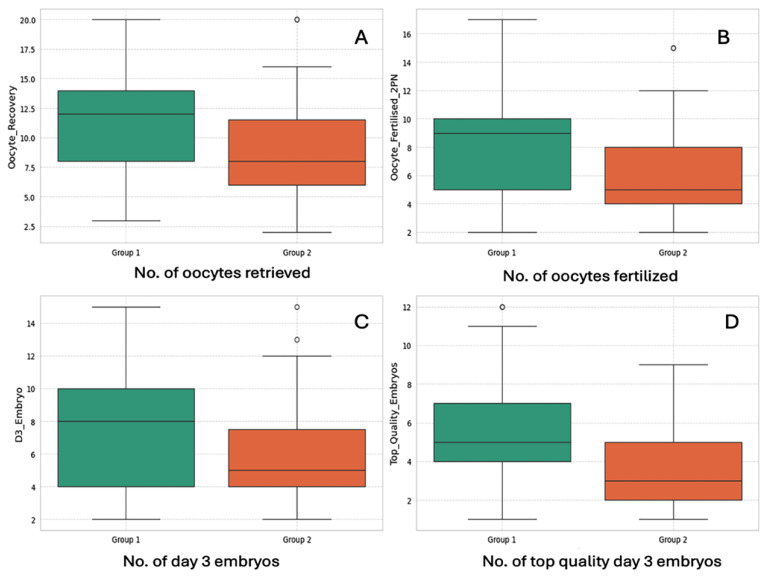
**Comparison of laboratory outcome measures between 2 groups.** Box and whisker plots showing that (**A**) number of oocytes/COCs retrieved, (**B**) Number of oocytes fertilized to 2PN stage, (**C**) number of day 3 cleavage stage embryos formed, and (**D**) number of top quality day 3 embryos generated; were all greater (*p* < 0.05) in Group 1 (Day 1 Antagonist, depicted in Green) in comparison to Group 2 (Day 5/day 6 Antagonist, depicted in Orange).

**Figure 3:**
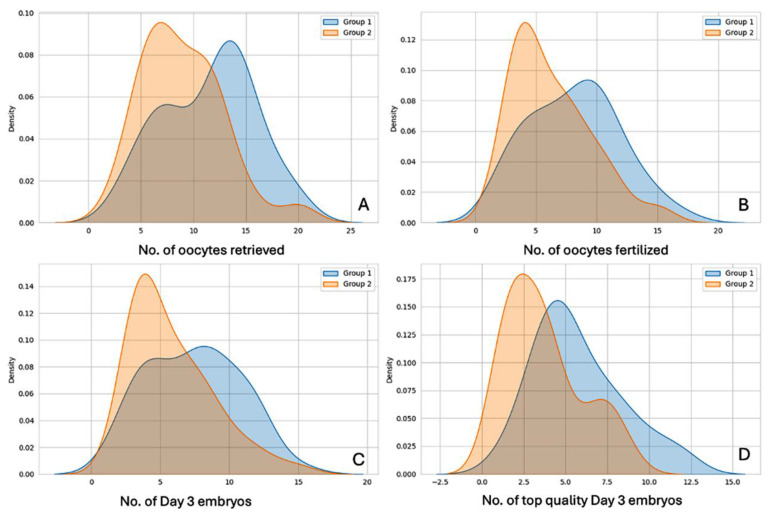
**Density distributions of laboratory outcome parameters compared between the 2 groups.** Distribution plots showing (**A**) number of oocytes/COCs retrieved, (**B**) Number of oocytes fertilized/2PN Number, (**C**) number of day 3 embryos formed, and (**D**) number of top quality embryos formed; compared between Group 1 (Day 1 Antagonist, in Blue) and Group 2 (Day 5/day 6 Antagonist, in orange).

Laboratory data are summarized in Table 2. Laboratory outcome measures such as number of oocytes/COCs retrieved, number of oocytes fertilized (2PN number), number of day 3 cleavage-stage embryos formed, and number of top-quality day 3 embryos generated were all significantly higher in the day 1 antagonist group (Group 1), as shown in Figure 2. The data distribution for all four variables are graphically presented in Figure 3.

The density distributions of the above four parameters are shown in Figure 3, for Group 1 (antagonists from day 1) vs. Group 2 (antagonists from day 5 or day 6).

However, it was likely that the number of fertilized oocytes (2PN number) and number of day 3 embryos formed would be greater in Group 1 as the number of oocytes retrieved and inseminated was significantly higher. Hence, for better interpretability, we compared the proportion of oocytes fertilized, as well as the proportion of top-quality day 3 cleavage-stage embryos generated (with total number of day 3 embryos formed used as the denominator) between the two groups. This serves as a more accurate marker of any potential difference in the quality of oocytes and embryos between the two groups, as shown in Table 3.

The results revealed that while there was no difference in the fertilization rates between the two groups, a greater proportion of top-quality cleavage-stage embryos were generated in patients who had received GnRH antagonist injections from day 1 of stimulation (Group 1).

### 3.2. Clinical Outcome Parameters

Finally, we compared the cumulative pregnancy outcomes over a total of three transfer cycles between the two groups (shown in Table 4). In Group 1 (antagonists from day 1), a total of 42 patients had a total of 50 embryo transfers over a maximum of three transfer cycles (maximum of one fresh and two frozen cycles), with a total of 91 embryos being transferred (mean number of embryos transferred per patient: 2.02 ± 0.92). In Group 2 (antagonists from day 5 or day 6), 55 patients had 68 embryo transfers (maximum of one fresh and two frozen cycles) with 124 embryos being transferred in total (mean of 2.27 ± 1.06 embryos transferred per patient). There was no significant difference between the mean number of embryos transferred between the two groups (*p* > 0.05).

**Figure 4:**
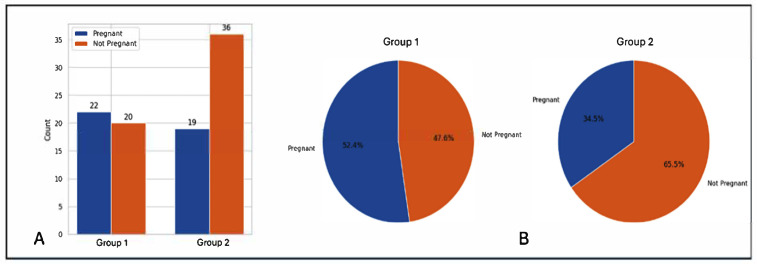
**Comparison of pregnancy outcomes between Group 1 (Day 1 Antagonist) and Group 2 (Day 5/6 Antagonist).** (**A**) Bar graph showing number of patients who became pregnant (blue) vs. number who did not get pregnant (orange), (**B**) Pie chart showing proportions of patients (Clinical pregnancy rate per stimulation cycle) who became pregnant (blue) vs those who did not (orange) in the 2 groups.

The cumulative clinical pregnancy rate (CPR) per embryo transfer (Group 1 vs. Group 2: 44% vs. 27.9%) and CPR per initiated stimulation cycle (52.4% vs. 34.5%) were higher for Group 1, although the differences were not statistically significant (*p* = 0.07). However, there was a clear trend of better pregnancy outcomes in the group with GnRH antagonist administration from day 1 of IVF stimulation. Pregnancy outcomes are summarized in Table 4 and represented graphically in Figure 4.

## 4. Discussion

### 4.1. Number of COCs Retrieved and Oocyte Fertilization Rate 

Baseline parameters such as patient age, BMI, AMH, and TSH levels were found to be comparable between Group 1 (antagonist from day 1 of stimulation) and Group 2 (antagonist from day 5 or 6 of stimulation). However, data analysis revealed that the number of oocytes (COCs) retrieved was significantly higher in the early antagonist group (Group 1 vs. Group 2: 11.47 ± 4.37 vs. 8.95 ± 3.88, *p* < 0.01). Both the groups, however, showed almost identical fertilization rates (Group 1 vs. Group 2: 70.9% vs. 70.1%, *p* > 0.05).

Interestingly, a recent 2024 retrospective study that was designed to solely compare oocyte yield between GnRH antagonist cycles with an added 3-day pretreatment course with a GnRH antagonist in the early follicular phase (“pretreatment cycle”), with a standard flexible antagonist protocol (“standard cycle”), also reported a significantly higher number of COCs retrieved in the pretreatment group [30].

It is well documented that ovarian follicles in PCOS patients are relatively refractory to the stimulatory effect of FSH. Follicular resistance to FSH signaling may be partly caused by intra-ovarian hyperandrogenism as well as elevated AMH levels. LH is known to have a stimulatory effect both on AMH secretion as well as on androgen synthesis from ovarian theca cells, acting synergistically with insulin. Indeed, serum LH levels are characteristically raised in a subset of PCOS patients, who often demonstrate altered LH pulse amplitude as well as pulse frequency.

Thus, GnRH antagonist-mediated suppression of LH overexpression in the early follicular phase of ovarian stimulation in Group 1 patients may have led to correction of AMH and androgen levels downstream in the HPO axis, ultimately resulting in improved ovarian follicular sensitivity to FSH and superior recruitment of preantral follicles. Unfortunately, due to the retrospective nature of this study, serum LH levels at various time points over the course of OS were not available uniformly across the two cohorts. This is also due to the fact that this was an unfunded study, and many of our patients cannot afford repeated serum hormone measurements, if not strictly clinically required.

### 4.2. Proportion of Top-Quality Embryos Achieved

Our data also shows that the proportion of top-quality day 3 cleavage-stage embryos obtained was significantly higher in the day 1 antagonist group compared to the flexible day 5/day 6 antagonist group (Group 1 vs. Group 2: 77.5% vs. 63.4%, *p* < 0.01). Since we had excluded all patients suffering from male factor infertility from this study, and baseline parameters (age, BMI, AMH, TSH, stimulation duration) were similar between the two groups, the only explanation for this finding appears to be one that implicates a difference in oocyte quality between the two groups.

It has been documented that PCOS patients have a higher risk of infertility even when ovulation is present [31]. Moreover, it has been suggested PCOS patients have relatively poor oocyte quality, which may be responsible for poorer outcomes in IVF cycles [32]. Given that elevated serum LH levels are a characteristic feature in many PCOS patients, and the fact that the only variable intervention in our study was the early administration of a GnRH antagonist from day 1 of stimulation in the study group, it is worthwhile to consider the possible impact of LH on oocyte quality in the light of our findings.

Elevated, premature serum LH levels in flexible antagonist IVF cycles have been found to have a detrimental impact on oocyte and embryo quality [33]. This is particularly true in the case of elevated serum LH levels early in the follicular phase, which has been proposed to result in the early resumption of meiosis and premature ovulation [34]. Moreover, it has been shown that in GnRH antagonist IVF cycles in women with PCOS, patients with higher basal serum LH levels exhibit an increased incidence of early LH elevation > 10 U/L [34]. Furthermore, in a remarkably granular analysis, the study by Wang et al. published in the Journal of Clinical Medicine in 2022 demonstrated that the proportion of top-quality embryos obtained was higher when the ratio of serum LH on the day of hCG administration (h-LH) to the basal serum LH value (b-LH) was greater than 1 (h-LH/b-LH > 1).

Thus, the available evidence seems to suggest that adequate suppression of basal LH with GnRH antagonists early from day 1 in the follicular phase of ovarian stimulation likely optimizes the meiotic pathway, improves oocyte quality, and leads to the generation of a higher proportion of top-quality embryos compared to the flexible initiation of GnRH antagonists from day 5 or day 6 of ovarian stimulation. 

### 4.3. Cumulative Clinical Pregnancy Rate 

Cumulative clinical pregnancy rates were analyzed over a maximum of three different embryo transfer cycles, including one fresh transfer cycle (in cases where a fresh transfer was performed) and up to two frozen transfer cycles. The mean number of embryos transferred was similar between the two groups (Group 1 vs. Group 2: 2.02 ± 0.92 vs. 2.27 ± 1.06, *p* > 0.5). The cumulative clinical pregnancy rate per embryo transfer (Group 1 vs. Group 2: 44% vs. 27.9%, *p* = 0.07), and cumulative clinical pregnancy rate per initiated cycle (Group 1 vs. Group 2: 52.4% vs. 34.5%, *p* = 0.07) showed higher trends in the day 1 antagonist group but just fell short of statistical significance.

This finding is similar to the results of the three-arm study previously cited (GnRH agonist vs. standard GnRH antagonist vs. early GnRH antagonist), which was designed to assess any differences between the three groups in terms of the number of oocytes retrieved, incidence of OHSS, and clinical pregnancy rate. The authors reported that although the number of oocytes retrieved was similar between the three groups, the incidence of OHSS was lower and clinical pregnancy rate was higher in the early antagonist group, although these differences did not achieve statistical significance [27].

A higher cumulative CPR has been reported in patients with better suppression of serum LH levels on the day of hCG [33]. Recent research has also implicated the combined impact of elevated serum LH, hyperandrogenism, and insulin resistance on the impairment of endometrial receptivity in PCOS patients [35]. We can only speculate that the trend of a higher cumulative CPR in our day 1 antagonist group was also secondary to the earlier and more effective suppression of serum LH throughout the follicular phase of ovarian stimulation, leading to a beneficial impact on oocyte quality, as well as endometrial receptivity.

## 5. Conclusions

To conclude, optimizing controlled ovarian stimulation is essential in IVF cycles to maximize oocyte yield and quality, while at the same time minimizing the risk of OHSS. This is particularly important in PCOS patients. Numerous studies have shown that GnRH antagonist protocols offer the best combination of cycle flexibility and a lower risk of OHSS, while offering acceptable IVF outcomes, compared with traditional long agonist protocols. However, there is little data comparing IVF outcomes with antagonist administration from day 1 of stimulation versus standard flexible antagonists. 

In this paper, we report a significantly higher oocyte yield and generation of top-quality embryos with the administration of antagonists from day 1 of IVF stimulation compared to standard flexible antagonists. We also documented higher cumulative pregnancy rates with this strategy over three embryo transfer cycles, both fresh and frozen, although this result just failed to achieve statistical significance. In the light of authoritative data which establishes the detrimental impact of early, continued, elevated serum LH levels on oocyte quality, as well as recent research which proposes the detrimental impact of early, sustained elevated LH levels on endometrial receptivity in PCOS patients [35], we speculate that these may be the underlying mechanisms that explain our findings. We propose that the early initiation of GnRH antagonists from day 1 of IVF stimulation for PCOS patients may help to optimize IVF outcomes. 

## 6. Limitations and Future Work

Our study is associated with certain limitations such as a relatively small sample size, retrospective design, and lack of funding. Larger, randomized, prospective studies should be conducted to attempt to replicate our findings and further optimize the care of PCOS patients desiring conception by IVF.

## Figures and Tables

**Table 1:** **Comparison of baseline parameters**. Analysis of Baseline Parameters of the 2 groups confirmed that the cohorts were well matched in terms of age and BMI of the participants; baseline AMH and TSH levels; as well as average number of days of ovarian stimulation, between the 2 groups. There was no statistical difference between the means of any of these parameters between the 2 groups.

Baseline Parameter	Group 1 (Day 1 Antagonist): Mean ± SD	Group 2 (Day 5/Day 6 Antagonist): Mean ± SD	*p* Value	Statistical Significance
Age (years)	29.13 ± 4.13	29.89 ± 4.55	>0.05	NS *
BMI (kg/m^2^)	24.48 ± 3.04	25.33 ± 3.16	>0.05	NS
AMH (ng/mL)	6.12 ± 2.31	5.61 ± 2.42	>0.05	NS
TSH (mIU/mL)	2.70 ± 1.29	3.09 ± 1.76	>0.05	NS
Average duration of stimulation (days)	10.6 ± 2.1	9.9 ± 1.9	>0.05	NS

* NS = Not significant.

**Table 2:** **Comparison of laboratory outcome measures**. Data analysis revealed that number of oocytes retrieved, number of oocytes fertilized, number of day 3 cleavage stage embryos formed, and number of top quality Day 3 embryos generated were all significantly greater (*p* < 0.05) in Group 1 (Day 1 antagonist) in comparison to Group 2 (Flexible standard antagonists from day 5 or 6 of stimulation).

Outcome Measure	Group 1 (Day 1 Antagonist): Mean ± SD	Group 2 (Day 5/Day 6 Antagonist): Mean ± SD	t-Value	*p* Value	Statistical Significance
No. of oocytes retrieved (COCs)	11.47 ± 4.37	8.95 ± 3.88	3.05	<0.01	S **
No. of oocytes fertilized (2PN Number)	8.13 ± 3.71	6.27 ± 3.20	2.69	<0.01	S
No. of day 3 cleavage-stage embryos formed	7.40 ± 3.33	5.82 ± 2.96	2.51	*p* = 0.01	S
No. of top-quality day 3 embryos	5.73 ± 2.63	3.69 ± 2.25	4.15	<0.01	S

** S = Significant.

**Table 3:** **Comparison between proportions of oocytes fertilized and top quality day 3 cleavage stage embryos formed between the 2 groups**. The data revealed that proportion of oocytes fertilized (fertilization rate) was very similar between the 2 groups; However, proportion of topr quality cleavage stage embryos obtained on day 3 was significantly greater (*p* < 0.05) in Group 1 (Day 1 antagonist), when compared to Group 2 (Day 5/day 6 antagonist).

Outcome Measure	Group 1 (Day 1 Antagonist)	Group 2 (Day 5/Day 6 Antagonist):	Z-Statistic	*p* Value	Statistical Significance
Proportion of oocytes fertilized (%)	0.709 (70.9%)	0.701 (70.1%)	0.28	>0.05	NS *
Proportion of day 3 embryos graded as top-quality embryos (%)	0.77 (77.5%)	0.63 (63.4%)	3.93	<0.01	S **

* NS = Not significant; ** S = Significant.

**Table 4:** **Embryo transfer and cumulative clinical pregnancy data from the 2 groups.** Although the number of embryos transferred was similar between the 2 groups (*p* > 0.05), both cumulative clinical pregnancy rate per stimulation cycle (Group 1 vs. Group 2: 52.4% vs. 34.5%), as well as cumulative clinical pregnancy rate per embryo transfer (Group 1 vs. Group 2: 44% vs. 27.9%) showed higher trends in the Day 1 antagonist group when compared with the day 5/day 6 antagonist group, although the difference just failed to achieve statistical significance (*p* = 0.07).

Study Groups	No. of Patients Who Had Embryo Transfer/s	No. of Embryo Transfer (ET) Events	Total No. of Embryos Transferred	No. of Pregnancies	Cumulative Clinical Pregnancy Rate (CPR) per Stimulation Cycle	Clinical Pregnancy Rate (CPR) per ET
Group 1 (day 1 antagonist)	42	50	91	22	52.4%	44%
Group 2 (day 5/day 6 antagonist)	55	68	124	19	34.5%	27.9%
Statistical significance			* NS *p* > 0.05		NS *p* = 0.07	NS *p* = 0.07

* NS: Not significant.

## Data Availability

Data is available upon reasonable request.

## Abbreviations

GnRH	gonadotropin-releasing hormone
PCOS	polycystic ovary syndrome
IVF	in vitro fertilization
FSH	follicle-stimulating hormone
LH	luteinizing hormone
PCOM	polycystic ovary morphology
AEPCOS	Androgen Excess and PCOS Society
AMH	anti-Mullerian hormone
HPO	hypothalamic–pituitary–ovarian
OS	ovarian stimulation
OHSS	ovarian hyperstimulation syndrome
FET	frozen embryo transfer
2PN	2 pronuclear
ASRM	American Society for Reproductive Medicine
GDIFR	Institute for Fertility Research
IRM	Institute of Reproductive Medicine
ICSI	intra-cytoplasmic sperm injection
CPR	clinical pregnancy rate
COC	cumulus–oocyte complex
IU	international unit
E2	estradiol
TVS	transvaginal sonography
r-hCG	recombinant human chorionic gonadotropin
HPI	hours post-insemination
ET	embryo transfer
HRT	hormone replacement therapy
MR	miscarriage rate
OPR	ongoing pregnancy rate
BMI	body mass index
TSH	thyroid-stimulating hormone
